# Spaceflight Effects on Cytochrome P450 Content in Mouse Liver

**DOI:** 10.1371/journal.pone.0142374

**Published:** 2015-11-11

**Authors:** Natalia Moskaleva, Alexander Moysa, Svetlana Novikova, Olga Tikhonova, Victor Zgoda, Alexander Archakov

**Affiliations:** Institute of Biomedical Chemistry, Moscow, Russia; Macau University of Science and Technology, MACAO

## Abstract

Hard conditions of long-term manned spaceflight can affect functions of many biological systems including a system of drug metabolism. The cytochrome P450 (CYP) superfamily plays a key role in the drug metabolism. In this study we examined the hepatic content of some P450 isoforms in mice exposed to 30 days of space flight and microgravity. The CYP content was established by the mass-spectrometric method of selected reaction monitoring (SRM). Significant changes in the CYP2C29, CYP2E1 and CYP1A2 contents were detected in mice of the flight group compared to the ground control group. Within seven days after landing and corresponding recovery period changes in the content of CYP2C29 and CYP1A2 returned to the control level, while the CYP2E1 level remained elevated. The induction of enzyme observed in the mice in the conditions of the spaceflight could lead to an accelerated biotransformation and change in efficiency of pharmacological agents, metabolizing by corresponding CYP isoforms. Such possibility of an individual pharmacological response to medication during long-term spaceflights and early period of postflight adaptation should be taken into account in space medicine.

## Introduction

Along with the growing knowledge about spaceflights it becomes obvious their influence on living organisms, including the human being. The "Bion" project of the Institute of Biomedical Problems of the Russian Academy of Sciences (IBMP RAS) includes a series of space orbital flights of animals on biosatellites and is aimed at identifying the cellular and molecular mechanisms of adaptation to microgravity. The information obtained can be used to ensure astronauts’ workability in long-term space flights, correcting both the diet and possible medical treatment.

The efficiency of the medication is known to be determined by the activity of the drug metabolism system. The enzymes of cytochrome P450 superfamily, which are an important part of the liver monooxygenase system, play a key role in the Phase I drug metabolism. Activity of the monooxygenase system towards a particular drug is mainly determined by concentrations of cytochrome P450 isoforms specific to it [1].

Mice are the most common objects for studies of the monooxygenase system as they ensure a holistic and physiologically adequate model to study the effects of various factors on the cytochrome P450 activity, inhibition and induction [2], [3].

Targeted proteomics was launched a decade ago with the use of selected reaction monitoring (SRM) in triple quadrupole detector [4]. Currently, this method is considered as a competitor of conventional immunoassays [5]. Usage of the stable isotope labeled peptide as an internal standard allows both the high confident identification and absolute quantitation of the targeted peptide. Besides, SRM allows multiplexing and may be used for simultaneous quantification of hundreds of proteins in many samples [6].

In the present work we examined the hepatic content of some P450 isoforms in the mice exposed to 30 days of space flight and microgravity using the mass-spectrometric method of SRM.

Therefore, the experiments on mice carried out in the framework of the “Bion-M1” project can be the basis for evaluating the spaceflight effects on the human monooxygenase system.

## Materials and Methods

The livers of 4–5 months old male mice (C57/BL6 line) of the flight group (SF, n = 4), the group of the ground control (GC, n = 4). The mice of the flight group were examined immediately after landing and after 7-days of postflight re-adaptation (RA, n = 3).

SF mice were exposed to microgravity for 30 days aboard the “Bion-M1” biosatellite during period from April 19 to May 19 2013. In the course of GC the experiment the group of animals was kept in the “Bion-M1” biosatellite blocks used in the flight experiment from July 26 to August 26, 2013. The blocks for animal alimentation were placed in a climatic chamber, where temperature, humidity and atmosphere gas composition were maintained the same as in the case animals of the biosatellite space flight. SF and GC mice were fed with the chow developed at IBMP RAS and it consisted of a paste of standard chow with water and casein as a gelling agent. More detailed information about conditions of the experiment can be found in the paper of Andreev-Andrievskiy *et al* [7].

Mice were euthanized by the method of cervical dislocation. The biomaterial was immediately taken from these animals and subjected to preprocessing for tissue analysis.

The study was approved by IACUC of MSU Institute of Mitoengineering (Protocol No–35, 1 November, 2012) and of Biomedical Ethics Commission of IBMP (protocol No–319, 4 April, 2013) and conducted in compliance with the European Convention for the Protection of Vertebrate Animals used for Experimental and Other Scientific Purposes.

### Sample preparation

Mice liver samples (20 mg each) were washed in cold 0.9% sodium chloride, homogenized in 200 μl of the lytic buffer (4% sodium deoxycholate in 0,1 M of Tris-HCl pH 8,5) and centrifuged at 3000 g at 4°C for 5 min.

The aliquot of liver protein (100 μg) was supplemented with a lysis buffer (4% SDS in 0.1 M Tris-HCl pH 8.5) to a volume of 30 μL and placed on the Microcon YM-10 filters (Millipore, USA) for the tryptic digestion according to the FASP protocol [8]. Briefly, the samples were supplemented with 100 μl of a reducing solution (0.1 M 1,4-dithiothreitol (DTT) in 0,1 M Tris-HCl pH 8,5), incubated for 40 min at 56°C, and centrifuged at 10 000 g for 15 min at 20°C. The samples were then washed 2 times with 200 μl of solution of 8 M urea in 0,1 M Tris-HCl, pH 8,5, followed by centrifugation at 10 000 g at 20°C for 15 min. The process of carbamidomethylation was carried out in 100 μl of 50 mM iodoacetamide (Sigma-Aldrich) in 0.1 M Tris-HCl, pH 8.5, for 30 min at room temperature. The samples were then washed 2 times with 200 μl of solution of 8 M urea in 0,1 M Tris-HCl, pH 8,5, followed by centrifugation at 10 000 g at 20°C for 15 min and 3 times with buffer for tryptic cleavage (50 mM tetraethylammonium bicarbonate pH 8.5). To carry out the enzymatic hydrolytic cleavage the sample was supplemented with 50 μl of buffer for tryptic cleavage containing trypsin (20 ng/μl) (Promega; Madison, WI, USA). After 16 hours of incubation at 37°C, the samples were supplemented with 50 μl of 30% formic acid, containing 200 fmoles of all isotope-labeled standards and centrifuged at 10 000 g for 10 minutes. Pass through fractions were used for further SRM analysis.

The protein content was measured by BCA method (Pierce, USA) according to the manufacturer's recommendations.

### Selection and synthesis of the prototypic peptide standards for quantitative analysis

Preliminary selection of prototypic peptides for quantitative analysis was carried out on the basis of the general proteomic profiling using the quadrupole-orbitrap mass-spectrometric detector “Q Exactive” connected to Ultimate 3000 RSLCnano HPLC (Dionex, Thermo Scientific).

Peptide separations were carried out using an Ultimate 3000 RSLCnano system (Dionex). The sample was loaded into a trap column (Acclaim PepMap, 2 cm × 75 μm inner diameter, C_18_, 3 μm, 100 A) (Thermo Scientific) at 2 μl/min with 0.1% formic acid in water. After 5 min, the trap column was set on-line with an analytical column (Zorbax 300SB- C_18_, 15 cm × 75 μm inner diameter, 3,5 μm) (Agilent). Peptide elution was performed by applying a mixture of solvents A and B. Solvent A was HPLC grade water with 0.1% (v/v) formic acid, and solvent B was 80%(v/v) HPLC grade acetonitrile/water with 0.1% (v/v) formic acid. Separations were performed by applying a linear gradient from 5% to 60% solvent B at 400 nl/min over 85 min followed by a washing step (10 min at 99% solvent B) and an equilibration step (25min at 5% solvent B).

MS analyses were performed using a Q-Exactive HF mass spectrometer (Thermo Scientific, Germany). A nanospray Flex ion source with 1800 V ionization voltage and 200°C capillary temperature were used. MS data were acquired in a data-dependent strategy selecting the fragmentation events based on the precursor abundance in the survey scan. MS survey scans were acquired at a resolution of 70000, scan range 400–1200 m/z, target AGC values of 1 × 10^6^, maximum injection time 50 ms. Fragmentation was performed for 25 most abundant ions with a normalized collision energy of 35, dynamic exclusion 10 s. MS/MS scans were acquired with resolution of 17500, target AGC values of 1 × 10^5^, maximum injection time 100 ms, isolation window 2,0 m/z.

### Internal Standard Production

The peptides desired were obtained using the solid-phase peptide synthesis on the Overture (Protein Technologies, USA) or Hamilton Microlab STAR devices according to the method published in Hood et al [9]. The isotope-labeled leucine (Fmoc-Leu-OH-13C6,15N) was used for isotope-labeled peptide synthesis instead of the unlabeled leucine (Fmoc-Leu-OH). The concentrations of the peptides synthesized were measured by the method of amino acids analysis with fluorescent signal detection of amino acids derived after peptides acidic hydrolysis [10].

The peptide stock solutions were diluted with 1% formic acid to obtain a concentration of peptides from 0.1 to 2000 fmol/μl.

### LC-SRM Analysis

The quantitative analysis of proteotypic tryptic peptides of cytochrome P450 was performed using mass-spectrometer “Agilent 6490” QQQ connected to “Agilent 1200” HPLC and equipped with the “Jet Stream” ionization source. The following parameters were used for the “Jet stream” ionization source: the temperature of the drying gas was 280°C, the pressure in the nebulizer was 18 psi, the flow of the drying gas was 14 l/min, and the voltage on the capillary - 3000V. Table 1 shows the optimized mass spectrometry parameters of the peptides quantitative analysis.

**Table 1:** Optimized mass spectrometric parameters of the peptides used for the quantitative analysis.

CYP	Peptide	RT (min)	Parent ion m/z	Fragment ions	Collision energy, V
1A1	VDMTPTYGLTLK	19.7	669.8^2+^	892.4(y_8_); 993.4(y_9_); 446.7(y_8_^2+^)	22
	VDMTPTYG*LTLK	19.7	673.4^2+^	1000.6(y_8_); 899.53(y_9_); 450.8(y_8_^2+^)	22
1A2	NSIQDITSALFK	27.1	669.0^2+^	1022.6 (y_9_); 894.5 (y_8_); 315.2 (b_3_)	20
	NSIQDITSA*LFK	27.1	672.4^2+^	1028.8 (y_9_); 900.8 (y_8_); 315.2 (b_3_)	20
2C29	NISQSFTNFSK	17.3	636.9^2+^	228.0 (b_2_); 1045.0 (y_9_); 830.4(y_7_)	20
2C29	GTTVITSLSSVLHDSK	22.9	549.1^3+^	587.3(y_11_^2+^); 643,8(y_12_^2+^); 743,9(y_14_^2+^)	15
	GTTVITSLSSV*LHDSK	22.9	551.2^3+^	590.5(y_11_^2+^); 647.4(y_12_^2+^); 747.0(y_14_^2+^)	15
2C39	FINLVPNNIPR	21.86	648.9^2+^	710.5 (y_6_); 809.5 (y_7_); 375.3 (b_3_)	20
	FIN*LVPNNIPR	21.86	652.4^2+^	710.5 (y_6_); 809.5 (y_7_); 375.3 (b_3_)	20
2C50,54	VQEEIEHVIGK	14.24	427.7^3+^	582.4(y_10_^2+^-H_2_O); 518.3 (y_9_^2+^-H_2_O); 208.5(y_4_)	10
	VQEEIEHVIG*K	14.24	430.2^3+^	586.4(y_10_^2+^-H_2_O); 522.3 (y_9_^2+^-H_2_O); 212.6(y_4_)	10
2D9	FGDIVPVNLPR	21.8	614.0^2+^	695.5(y_6_); 320.1(b_3_); 794.3(y_7_)	19
	FGDIVPVN*LPR	21.8	617.4^2+^	702.4(y_6_); 320.1(b_3_); 801.5(y_7_)	19
2D10	FGDIAPLNLPR	21.9	607.0^2+^	709.3(y_6_); 780.1(y_7_); 320.1(b_3_)	18
	FGDIAPLN*LPR	21.9	610.4^2+^	716.4(y_6_); 787.5(y_7_); 320,1(b_3_)	18
2D26	DLTDAFLAEVEK	25.3	676.0^2+^	575.2(y_5_); 835.5(y_7_); 906.6(y_8_)	20
	DLTDAF*LAEVEK	25.3	679.4^2+^	575.2(y_5_); 842.5(y_7_); 913,6(y_8_)	20
2E1	DIDLSPVTIGFGSIPR	26.6	844.1^2+^	1143.4(y_11_); 344.1(b_3_); 733.1(y_7_)	30
	DID*LSPVTIGFGSIPR	26.6	847.5^2+^	1143.4(y_11_); 344.1(b_3_); 733.1(y_7_)	30
2E1	MNMPYMDAVVHEIQR	24.6	612.1^3+^	729.4(y_12_^2+^); 794.9(y_13_^2+^); 682.4(y_5_)	17
3A11,41	ALLSPTFTSGK	17.4	561.5^2+^	737.4(y_7_); 824.4(y_8_); 937.2(y_9_)	10
	AL*LSPTFTSGK	17.4	564.9^2+^	737.4(y_7_); 824.4(y_8_); 944.4(y_9_)	10
3A11,16	GSIDPYVYLPFGNGPR	26.1	584.8^3+^	744.4(y_7_); 372.2(b_4_); 895.3(b_8_)	11
	GSIDPYVY*LPFGNGPR	26.1	586.9^3+^	744.4(y_7_); 372.2(b_4_); 895.3(b_8_)	11
3A11,16	FALMNMK	19.1	427.8^2+^	636.2(y_5_); 707.2(y_6_); 523.2(y_4_)	8

* L—isotopically labeled leucine residue (Leu C_6_^13^N_1_^15^)

Chromatographic separation was carried out using the “Agilent 1200” HPLC including capillary pump and autosampler. Peptides separation was carried out using the analytical column Eclipse XDB C18, 100 x 3 mm, 3.5 um. Mobile phase was 0.1% formic acid in water; B is 0.1% formic acid in 80% acetonitrile; the flow rate was 100μl/min. The gradient of the mobile phase was from 5% B to 60% B for 40 min, then to 100% B to 44 minutes, then back to 5% B at 46 min.

### Data processing

Each SRM experiment was repeated in 3 technical runs. The results were manually inspected using Skyline software [11] to find transitions that were similar to those in the target peptides. For interference screening we applied the criteria described in Percy at al [12]. Briefly, the peptide was considered to be detected in the run if the differences between relative intensities for three transitions of endogenous and isotopically labeled peptide did not exceed 25% in the run and the transition chromatographic profiles of endogenous peptide were identical to the corresponding transitions of stable-isotope labeled peptide.

Calibration curves were obtained for each of the desired peptides using the mixtures of purified synthetic native peptides in the concentration range of 100 fmole/μl – 100 afmole/μl and its isotopically labeled analogues were added at the concentration of 2 fmole/μl. All calibration curves were linear in the range of 100 fmole/μl – 0.1 fmole/μl and showed the coefficient of linear regression equal to 0.95.

Prior to the sample processing, the performance of the LC-SRM platforms used was validated by obtaining the calibration curves of the corresponding set of SIS and synthetic natural peptides. Moreover, after five LC-SRM runs we verified the relevance of calibration by analyzing one of the calibration peptide solution at 10 fmole/μl. The data on precision and the accuracy of the method is presented in the S1 File.

The detection limit was defined as the lowest concentration determined on the linear part of calibration curve. It varies for different peptides in the range from 100 amole/μl to 200 amole/μl.

Labeled/unlabeled peptide peak area ratios were used to calculate the concentration of the targeted peptide in a sample.

$$\begin{array}{l} {\text{C}}_{\text{pept}}={\text{C}}_{\text{lab}}*{\text{S}}_{\text{pept}}{\text{/S}}_{\text{lab}}\text{ ,}\\ \text{Where:}\\ {\text{C}}_{\text{pept}}\text{ - target peptide concentration,}\\ {\text{C}}_{\text{lab}}\text{ - labeled peptide concentration,}\\ {\text{S}}_{\text{pept}}\text{ - area of target peptide peak,}\\ {\text{S}}_{\text{lab}}\text{ - area of labeled peptide peak}. \end{array}$$

Statistical analysis was performed using IBM^®^ SPSS^®^ Statistics V22.0 software. Kruskal–Wallis one-way analysis of variance was used to assess statistical significance for multiple experimental groups. For proteins with p-value<0.05 (1A1, 1A2, 2E1, 2D9 and 2C29) the pair-wise comparisons were performed by non-parametric Mann—Whitney U-test. P-value = 0.029 for SF/GC comparison and p-value = 0.057 for RA/GC and RA/SF comparisons was considered as statistically significant.

## Results and Discussion

The biological data on the adaptation of various systems of the body to weightlessness and re-adaptation to Earth’s gravity are needed to solve the problems of fast recovery of the astronauts’ performance after a long-term stay in space. The study of the influence of a space flight on content in the liver cytochromes P450 as key enzymes of the first phase of drug metabolism may help to optimize pharmacotherapy. Such studies on humans are often impossible, hence, the need for biomedical research on animals with the ultimate goal of providing a scientific substantiation and recommendations for the development of a system to prevent and correct the negative impact of a long-term space flight.

Mouse is one of the biological animal models, providing useful information about drug biotransformation. The mouse genome contains 36 orthologous genes of the cytochrome P450 family. Many of mouse P450 substrate specificities and inhibition patterns are similar to human cytochromes [13], as well as molecular mechanisms of their induction [14, 15].

For quantitative analysis of cytochrome P450, 11 samples of mouse livers were used. Four samples belonged to the space flight group (SF), four samples were ground control (GC) and three samples were used for recovery group (RA).

Concentrations of selected cytochrome P450 isoforms were measured using a triple quadrupole mass-spectrometer operated in SRM mode. This method is widely used for the quantitative analysis of target proteins of complex samples, since it provides a high sensitivity, reproducibility, and selectivity in a wide range of concentrations of [16–18]. Several research groups have used SRM to study cytochrome P450 in human and animal tissues. Cytochrome P450 is characterized by a high homology within the family or even between families. SRM analysis makes it possible to analyze prototypic peptides allowing isoform specific quantitative analysis of P450 in complex biological samples [19–21].

To determine proteotypic peptides for the targeted cytochrome P450 isoform we carried out shotgun proteomic profiling of mouse liver samples. According to obtained results, 3091 proteins were identified, 31 of which are belonged to the superfamily of cytochrome P450 (1A1, 1A2, 2A4, 2A5, 2A12, 2B10, 2E1, 2F2, 2J5, 4V2, 4A14, 4A10, 4A12A, 2D9, 2D10, 2D11, 2D26, 3A11, 3A13, 3A16, 3A25, 3A41, 2C29, 2C39, 2C38, 2C40, 2C54, 2C37, 2C55, 2C70, 4V2) (data not shown). Prototypic peptides of 10 most pharmacologically important P450 isoforms (CYP2E1, CYP1A1, CYP1A2, CYP3A5, CYP2C29, CYP2C37, CYP2C39, CYP2D9, and CYP2D10) were chosen for further quantitative SRM method development.

Totally, 14 peptides corresponding to ten CYP isoforms were chosen for protein identification and concentration determination by SRM. Eleven isotopically labelled internal standards, as well as corresponding non-labeled peptides were synthesized (plus 3 peptides were synthesized as unlabeled only). Table 1 shows the list of synthetic peptides used as internal or external standards. Parent ions and three corresponding fragment ions were determined for each proteotypic peptide. The procedure of SRM method developing is described in details in [22]. The linear calibration curves of the cytochrome peptides of selected P450 isoforms were built using the synthesized peptides in the concentration range from 0.1 to 100 fmol/μl. All complete experiments (from sample preparation to SRM measurements) were repeated at least three times. SRM is a powerful proteomic method for quantitative measurement of proteins. Usage of the stable isotope labeled peptide as an internal standard allows both the high confident identification and absolute quantitation of the targeted peptide [23].

**Fig 1:**
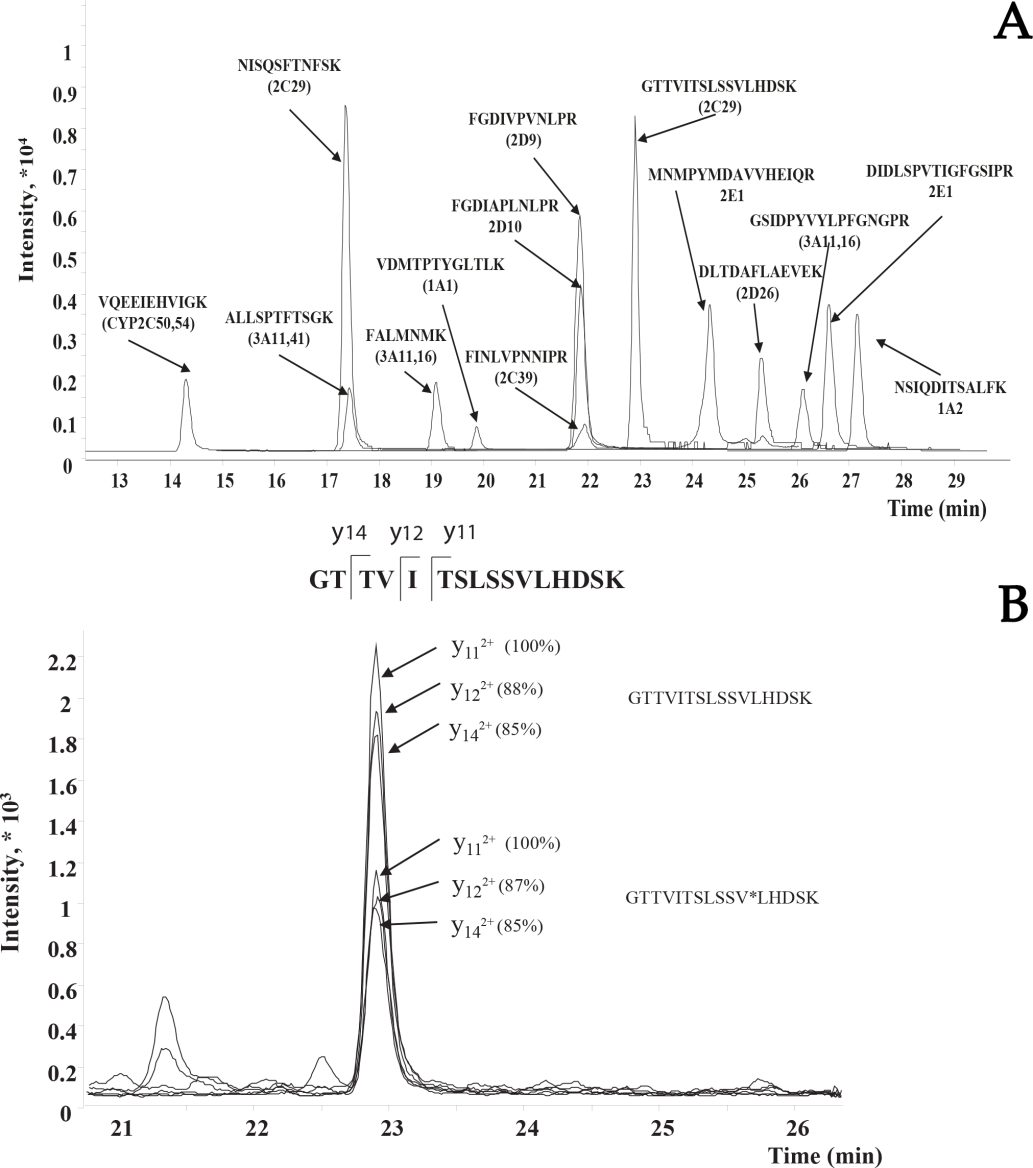
Extracted ion chromatogram of the target CYP peptides from the mouse liver digest obtained on LC-SRM-MS platform. (A) Single SRM transition is shown for each target peptide. (B) Three SRM fragment ion pairs for SIS and natural peptide are shown for peptide GTTVITSLSSVLHDSK from CYP2C29 (retention time 22.9 min). The relative ion ratios are indicated in parentheses.

Illustrative LC-SRM analysis of the chosen CYP isoforms peptide standards is shown in Fig 1A. As it is shown in Fig 1 some peptides are co-eluted at the reverse phase chromatography. However the separation of ions in MS allows distinguishing the signals from different peptides.

Validation of the SRM data obtained was performed using the criteria described in Percy at al [12]. As it is shown in Fig 1b, the differences between the relative intensities for three transitions of endogenous and isotopically labeled peptide did not exceed 2% in the run. The chromatographic profiles of endogenous peptide are identical to the corresponding stable-isotope labeled peptide. Finally, the concentration measured within 3 technical runs for this peptide had CV < 5%.

Table 2 shows the measured concentrations of cytochrome P450 in the mouse livers of the flight group (SF), ground control (GC), and recovery group (RA). The concentration of different cytochromes P450 isoforms varied from 68 fmol/μg to 0.46 fmol/μg for CYP2C29 and CYP 1A1, respectively. These values for the liver microsomal fraction are consistent with the data reported by Hersman and Bumpus [24]. A obvious increase of the CYP concentrations in the flight experiment, compared to the ground control experiment was observed for the CYP2E1 (1.8 times), CYP2C29 (1.4 times) and CYP1A2 (1.9 times). After the 7 days of recovery period the content of CYP 2C29 fell to initial value, 2D9 decreased in comparison with control level while CYP 2E1 level remained elevated. At the same time some negligible changes in CYP contents were observed between SF and RA conditions for 1A2, 1A1 and 2D9. The highest inter-individual variability of tested CYPs content in the mouse livers of the ground control experiment did not exceed 32% excluding 2D9 (46%), while in the flight experiment it was, 60% for 3A11, 3A16, 3A41 and 78% for 2C39. Therefore, the increase of 3 CYPs content as well as increase of inter-individual variability is associated with the influence of space flight conditions.

**Table 2:** Concentrations of cytochrome P450 in the mouse livers of the flight group (SF), ground control (GC), and recovery group (RA).

CYP	SF, fmol\μg of total protein^a^	GC, fmol\μg of total protein^a^	RA, fmol\μg of total protein^a^
1A1	0.53 ± 0.04	0.46 ± 0.12	0.78 ± 0.10**, ***
1A2	11.18 ±0.65*	5.94 ± 1.79	2.45 ± 1.59***
2D9	9.90 ± 2.40	7.10 ± 3.23	2.34 ±1.54**,***
2D10	11.40 ± 2.10	13.28 ± 1.28	11.31 ± 3.98
2D26	11.06 ± 1.82	13 ± 3.28	14.94 ± 8.70
2E1	33.45 ± 3.62*	18.51 ± 3,43	35.72 ± 3.13**
3A11,16	7.85 ± 4.37	7.97 ± 1.07	6.03 ± 0.91
3A11,41	4.46 ± 2.68	5.96 ± 0.32	7.62 ± 4.08
2C29	67.89 ± 5.10*	46.92± 7.09	40.83 ± 9.33***
2C39	2.26 ± 1.77	1.66 ± 0.54	4.44 ± 0.56
2C50,54	21.15 ± 5.7	14.41 ± 3.89	16.41± 2.91

^a^ means±SE of 4 separate experiments for SF and GC conditions and 3 separate experiments for RA condition

* statistically significant difference between SF and GC groups (p-value < 0.05)

** statistically significant difference between RA and GC groups (p-value < 0.1)

*** statistically significant difference between SF and RA groups (p-value < 0.1)

Liver is the major organ involved in drug biotransformation. The highest expressed forms in mouse liver are CYPs 2C29, 2C50/54, 2E1, while 2D, 3A subfamily members are less abundant. The Cyp1A subfamily members had different expression profiles across organs. Cyp1a2 was generally found in liver and Cyp1a1 is mainly expressed extrahepatically (highest mRNA expression found in lung and duodenum) [25]. Variation of P450 content in the liver and CYP genetic polymorphisms are two major sources of variability in drug pharmacokinetics and response. Among 105 putatively expressed in mice CYPs only enzymes of CYP1, 2, and 3 families are involved in the metabolism of 80% of all drugs in clinical use [26].

The cytochrome P450 system in humans and animals is involved in oxidation of numerous endogenous and exogenous compounds. These enzymes play an important role in the hydroxylation of steroid hormones, bile acids and cholesterol; oxidation of saturated and unsaturated fatty acids, biosynthesis of prostaglandins; oxidation of xenobiotics (medicines, poisons, drugs) and bioactivation of carcinogenic compounds (nitrosamines, aromatic hydrocarbons.

Elevation of some CYP content during and after space flight can lead to increase of corresponding substrates (medicines) clearance and, correspondently, decrease of pharmacological effects. In the case of CYP2E1, which content was doubled during the flight we could expect decrease of effect of alcohol or nitrosamines which are general substrates for this P450 isoform. CYP1A2 oxidizes such substrates as caffeine, aminophylline, paracetamol, estradiol, and theophylline. CYP2C forms are the main enzymes of biotransformation of nonsteroidal anti-inflammatory drugs (diclofenac, piroxicam, naproxen, indomethacin, and so on), proton pump inhibitors (omeprazole, lansoprazole) [1].

## Conclusion

Hard conditions of spaceflight such as stress, radiation and weightlessness is an extreme situation that can cause a number of physiological changes in the human body. Alterations in cardiovascular, immunological, sensorimotor, musculoskeletal and reproductive systems have been reported following the spaceflight [27–29]. Sometimes such changes can lead to the development of real disease which requires therapeutically medical treatment. In these cases the possibility of an individual pharmacological response to medication in long-term spaceflights, as well as after the flight should be taken into account in space medicine.

## Supporting Information

S1 File SRM method validation. (DOC)

## References

[1] Zanger UM, Schwab M. Cytochrome P450 enzymes in drug metabolism: regulation of gene expression, enzyme activities, and impact of genetic variation. Pharmacol Ther. 2013;138: 103–41. 10.1016/j.pharmthera.2012.12.007 23333322

[2] Muruganandan S, Sinal CJ. Mice as clinically relevant models for the study of cytochrome P450-dependent metabolism. Clin Pharmacol Ther. 2008;83: 818–28. 10.1038/clpt.2008.50 18388875

[3] Turpeinen M, Ghiciuc C, Opritoui M, Tursas L, Pelkonen O, Pasanen M. Predictive value of animal models for human cytochrome P450 (CYP)-mediated metabolism: a comparative study in vitro. Xenobiotica. 2007;37: 1367–77. 10.1080/00498250701658312 17943662

[4] Anderson L, Hunter CL. Quantitative mass spectrometric multiple reaction monitoring assays for major plasma proteins. Mol Cell Proteomics. 2006;5: 573–88. 10.1074/mcp.M500331-MCP200 16332733

[5] Archakov A, Lisitsa A, Ponomarenko E, Zgoda V. Recent advances in proteomic profiling of human blood: clinical scope. Expert Rev Proteomics. 2015;12: 111–3. 10.1586/14789450.2015.1018895 25711363

[6] Ponomarenko EA, Kopylov AT, Lisitsa A V, Radko SP, Kiseleva YY, Kurbatov LK, et al Chromosome 18 transcriptoproteome of liver tissue and HepG2 cells and targeted proteome mapping in depleted plasma: update 2013. J Proteome Res. 2014;13: 183–90. 10.1021/pr400883x 24328317

[7] Andreev-Andrievskiy A, Popova A, Boyle R, Alberts J, Shenkman B, Vinogradova O, et al Mice in Bion-M 1 space mission: training and selection. PLoS One. 2014;9: e104830 10.1371/journal.pone.0104830 25133741 PMC4136787

[8] Wiśniewski JR, Zougman A, Nagaraj N, Mann M. Universal sample preparation method for proteome analysis. Nat Methods. 2009;6: 359–62. 10.1038/nmeth.1322 19377485

[9] Hood CA, Fuentes G, Patel H, Page K, Menakuru M, Park JH. Fast conventional Fmoc solid-phase peptide synthesis with HCTU. J Pept Sci. 2008;14: 97–101. 10.1002/psc.921 17890639

[10] Fekkes D. State-of-the-art of high-performance liquid chromatographic analysis of amino acids in physiological samples. J Chromatogr B Biomed Appl. 1996;682: 3–22. Available: http://www.ncbi.nlm.nih.gov/pubmed/8832421. 8832421 10.1016/0378-4347(96)00057-6

[11] MacLean B, Tomazela DM, Shulman N, Chambers M, Finney GL, Frewen B, et al Skyline: an open source document editor for creating and analyzing targeted proteomics experiments. Bioinformatics. 2010;26: 966–8. 10.1093/bioinformatics/btq054 20147306 PMC2844992

[12] Percy AJ, Chambers AG, Smith DS, Borchers CH. Standardized protocols for quality control of MRM-based plasma proteomic workflows. J Proteome Res. 2013;12: 222–33. 10.1021/pr300893w 23245390

[13] Nelson DR, Zeldin DC, Hoffman SMG, Maltais LJ, Wain HM, Nebert DW. Comparison of cytochrome P450 (CYP) genes from the mouse and human genomes, including nomenclature recommendations for genes, pseudogenes and alternative-splice variants. Pharmacogenetics. 2004;14: 1–18. Available: http://www.ncbi.nlm.nih.gov/pubmed/15128046. 15128046 10.1097/00008571-200401000-00001

[14] Court MH, Von Moltke LL, Shader RI, Greenblatt DJ. Biotransformation of chlorzoxazone by hepatic microsomes from humans and ten other mammalian species. Biopharm Drug Dispos. 1997;18: 213–26. Available: http://www.ncbi.nlm.nih.gov/pubmed/9113344. 9113344 10.1002/(sici)1099-081x(199704)18:3<213::aid-bdd15>3.0.co;2-0

[15] Bogaards JJ, Bertrand M, Jackson P, Oudshoorn MJ, Weaver RJ, van Bladeren PJ, et al Determining the best animal model for human cytochrome P450 activities: a comparison of mouse, rat, rabbit, dog, micropig, monkey and man. Xenobiotica. 2000;30: 1131–52. 10.1080/00498250010021684 11307970

[16] Walther TC, Mann M. Mass spectrometry-based proteomics in cell biology. J Cell Biol. 2010;190: 491–500. 10.1083/jcb.201004052 20733050 PMC2928005

[17] Ludwig C, Claassen M, Schmidt A, Aebersold R. Estimation of absolute protein quantities of unlabeled samples by selected reaction monitoring mass spectrometry. Mol Cell Proteomics. 2012;11: M111.013987 10.1074/mcp.M111.013987 22101334 PMC3316728

[18] Zgoda VG, Kopylov AT, Tikhonova O V, Moisa AA, Pyndyk N V, Farafonova TE, et al Chromosome 18 Transcriptome Profiling and Targeted Proteome Mapping in Depleted Plasma, Liver Tissue and HepG2 Cells. J Proteome Res. 2013;12: 123–134. 10.1021/pr300821n 23256950

[19] Yu A-M, Qu J, Felmlee MA, Cao J, Jiang X-L. Quantitation of human cytochrome P450 2D6 protein with immunoblot and mass spectrometry analysis. Drug Metab Dispos. 2009;37: 170–7. 10.1124/dmd.108.024166 18832475 PMC2683657

[20] Langenfeld E, Zanger UM, Jung K, Meyer HE, Marcus K. Mass spectrometry-based absolute quantification of microsomal cytochrome P450 2D6 in human liver. Proteomics. 2009;9: 2313–23. 10.1002/pmic.200800680 19402041

[21] Kawakami H, Ohtsuki S, Kamiie J, Suzuki T, Abe T, Terasaki T. Simultaneous absolute quantification of 11 cytochrome P450 isoforms in human liver microsomes by liquid chromatography tandem mass spectrometry with in silico target peptide selection. J Pharm Sci. 2011;100: 341–52. 10.1002/jps.22255 20564338

[22] Moskaleva NE, Zgoda VG, Archakov AI. [Mass-spectrometric measurements of P450 isoform specific content and corresponding enzyme activities]. Bioorg Khim. 37: 149–64. Available: http://www.ncbi.nlm.nih.gov/pubmed/21721249. 21721249 10.1134/s1068162011010122

[23] Lange V, Picotti P, Domon B, Aebersold R. Selected reaction monitoring for quantitative proteomics: a tutorial. Mol Syst Biol. 2008;4: 222 10.1038/msb.2008.61 18854821 PMC2583086

[24] Hersman EM, Bumpus NN. A targeted proteomics approach for profiling murine cytochrome P450 expression. J Pharmacol Exp Ther. 2014;349: 221–8. 10.1124/jpet.113.212456 24594750 PMC3989799

[25] Renaud HJ, Cui JY, Khan M, Klaassen CD. Tissue distribution and gender-divergent expression of 78 cytochrome P450 mRNAs in mice. Toxicol Sci. 2011;124: 261–77. 10.1093/toxsci/kfr240 21920951 PMC3216415

[26] Hrycay EG, Bandiera SM. Expression, function and regulation of mouse cytochrome P450 enzymes: comparison with human P450 enzymes. Curr Drug Metab. 2009;10: 1151–83. Available: http://www.ncbi.nlm.nih.gov/pubmed/20166999. 20166999 10.2174/138920009790820138

[27] Zhu H, Wang H, Liu Z. Effects of real and simulated weightlessness on the cardiac and peripheral vascular functions of humans: A review. Int J Occup Med Environ Health. 2015;28: 793–802. 10.13075/ijomeh.1896.00301 26224491

[28] Aponte VM, Finch DS, Klaus DM. Considerations for non-invasive in-flight monitoring of astronaut immune status with potential use of MEMS and NEMS devices. Life Sci. 2006;79: 1317–33. 10.1016/j.lfs.2006.04.007 16757003

[29] Mark S, Scott GBI, Donoviel DB, Leveton LB, Mahoney E, Charles JB, et al The impact of sex and gender on adaptation to space: executive summary. J Womens Health (Larchmt). 2014;23: 941–7. 10.1089/jwh.2014.4914 25401937 PMC4236030

