# Supplementary material for: Spaceflight Effects on Cytochrome P450 Content in Mouse Liver
Source: PLoS One. 2015 Nov 11;10(11):e0142374. doi: 10.1371/journal.pone.0142374 (PMC4641588; doi:10.1371/journal.pone.0142374)
Supplement: S1 File — (DOC) [file pone.0142374.s001.doc]

**S1 file. SRM method validation**

The SRM data was processed with the Agilent MassHunter Quantitative Analysis software (version B.05.00 SP02). The data was processed to verify the peak selection and integration before the samples were analyzed for peak area measurements and for the generation of calibration curves. The relative responses of native/heavy peptides were used for the analyses. To determine the protein concentrations, the calibration curves were constructed with 7 concentration levels (with 5 replicates/level). The calibration curves were obtained for each of the desired peptides using the mixtures of purified synthetic native peptides in the concentration range of 100 fmol/µl – 100 afmol/µl and its isotopically labeled analogues were added at the concentration of 2 fmol/µl. All calibration curves were linear in the range of 100 fmol/µl – 0.1 fmol/µl and showed the coefficient of linear regression equal to 0.95. Each calibration curves were fitted to 1/y weighted linear regression model. The linear regression analysis (1/y weighted) for a peptide illustrates the dynamic range of the assay, and the error bars indicate the standard deviation of the measurements. The data points within the given concentration level had to be both precise (with an average CV of <20%) and obey the outlier determination requirement (80-120% on average).

In further validation of the method the precision and accuracy were determined by the analysis of standard samples with concentrations of 0.5, 5 and 50 fmol/µl using five determinations per concentration. The mean accuracy value was within 15% of the nominal value ( 85 - 115% nominal value). The precision ( CV) determined at each concentration did not exceed 15%.


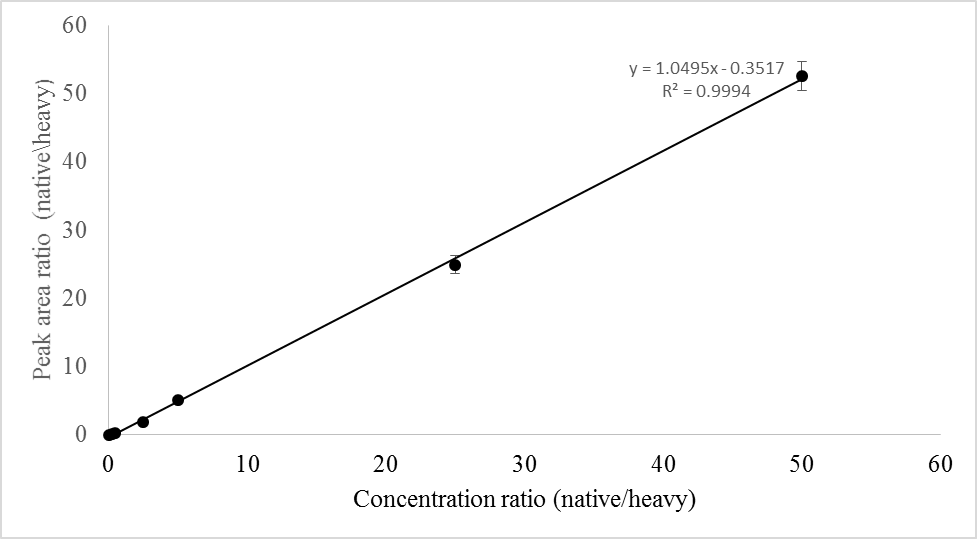


Fig 1. Calibration curve for VDMTPTYGLTLK (1A1)


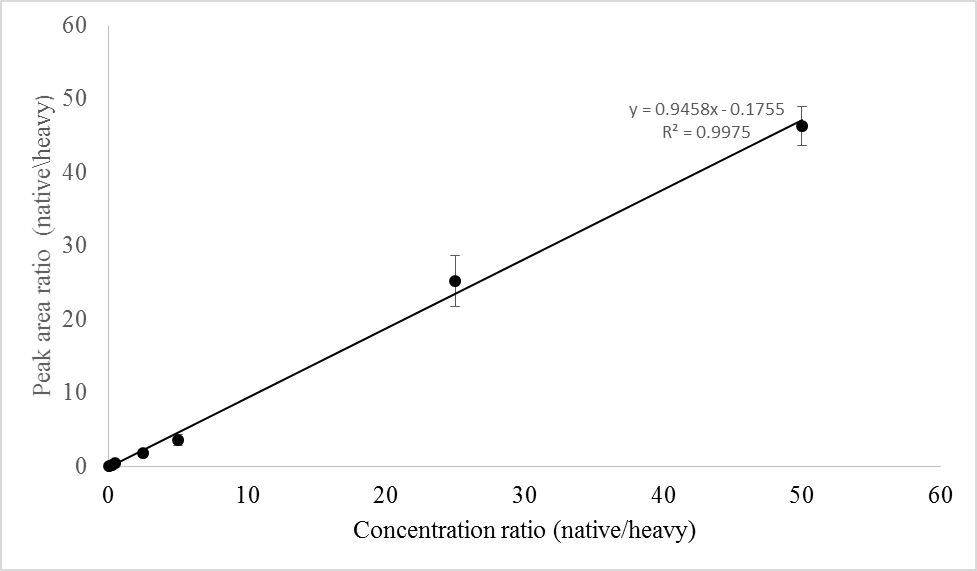


Fig 2. Calibration curve for NSIQDITSALFK (1A2)


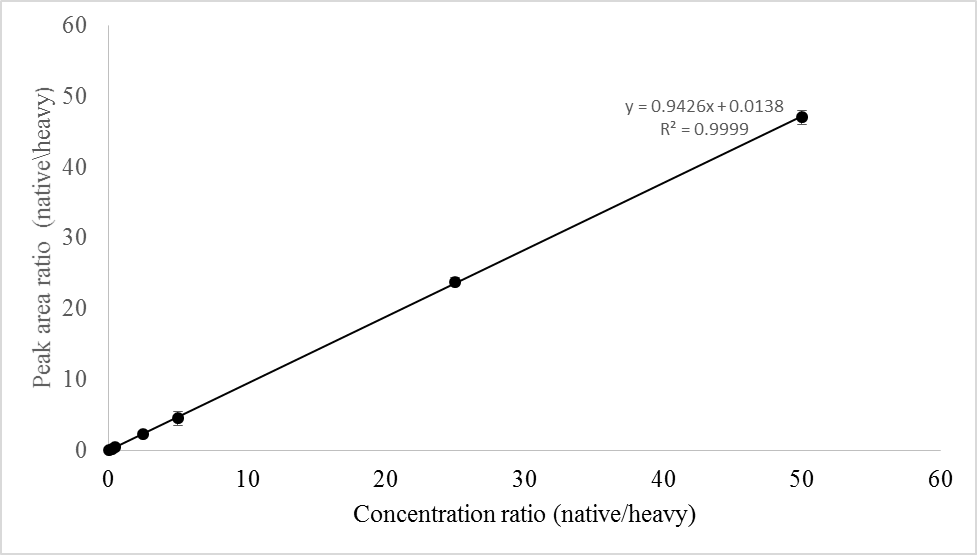


Fig 3. Calibration curve for GTTVITSLSSVLHDSK (2C29)


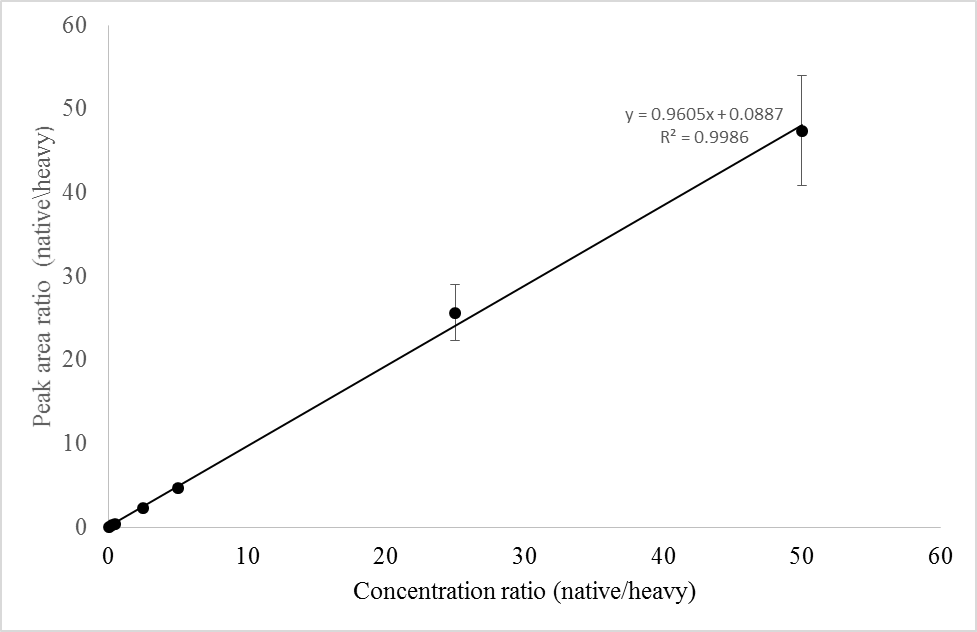


Fig 4. Calibration curve for FINLVPNNIPR (2C39)


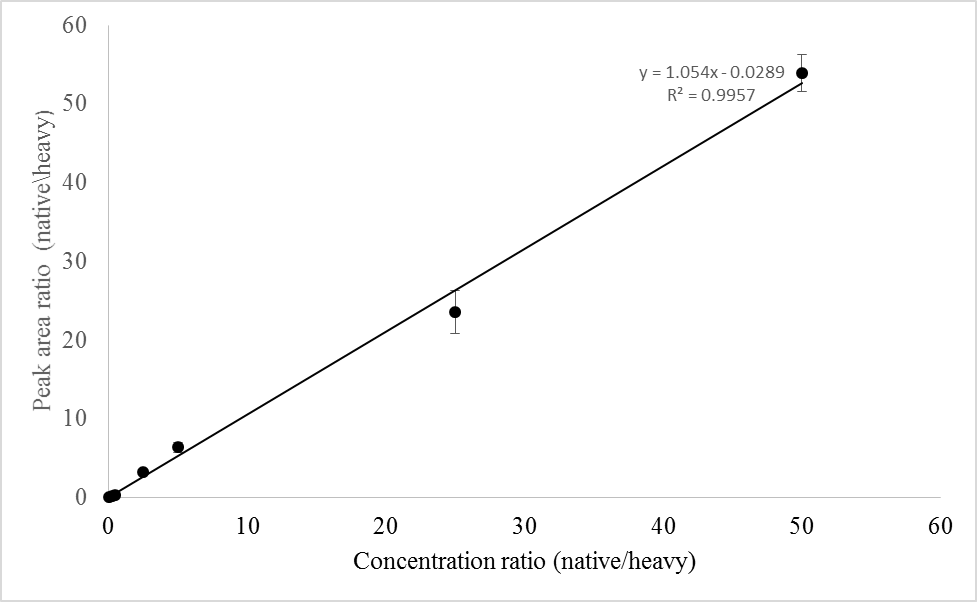


Fig 5. Calibration curve for VQEEIEHVIGK (2C50,54)


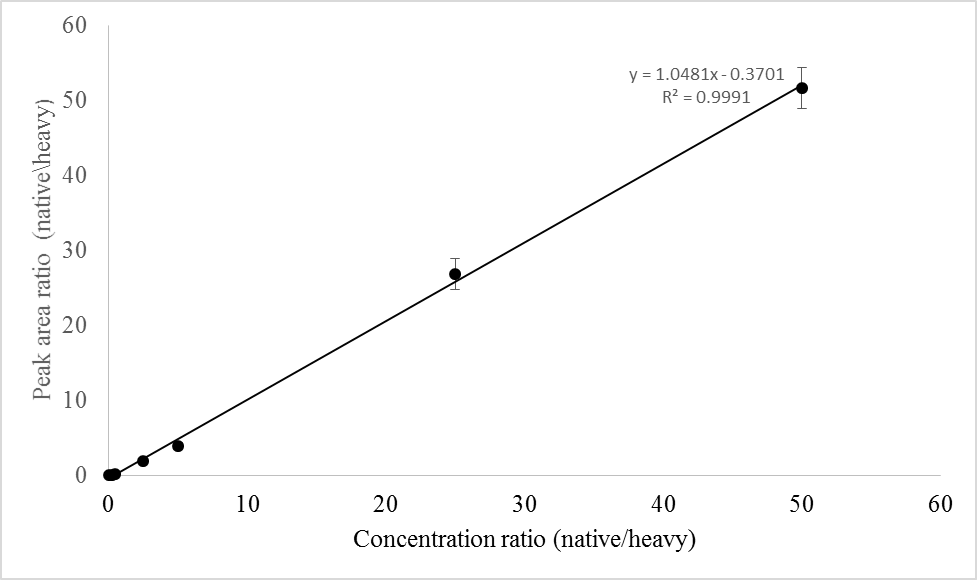


Fig 6. Calibration curve for FGDIVPVNLPR (2D9)


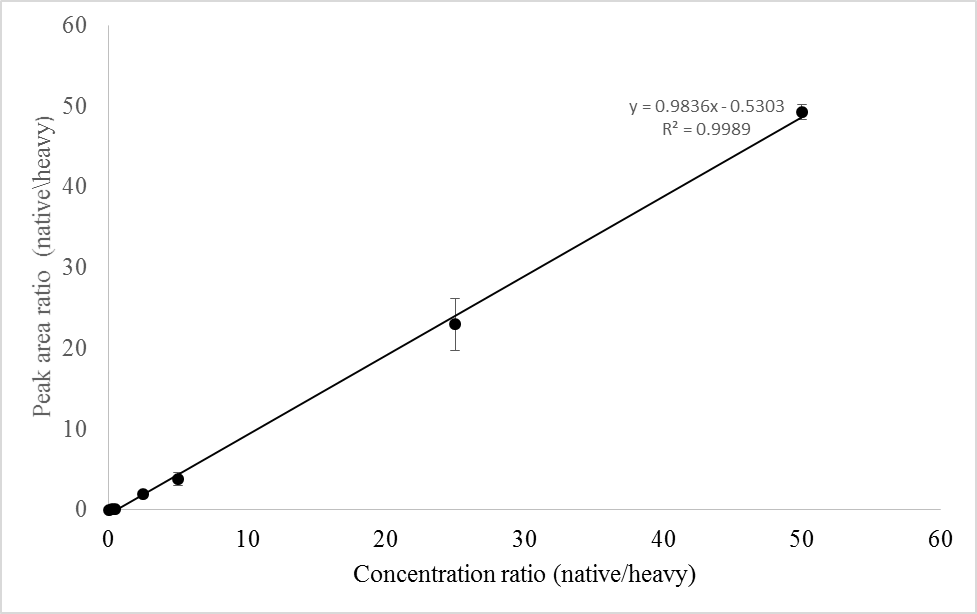


Fig 7. Calibration curve for FGDIAPLNLPR (2D10)


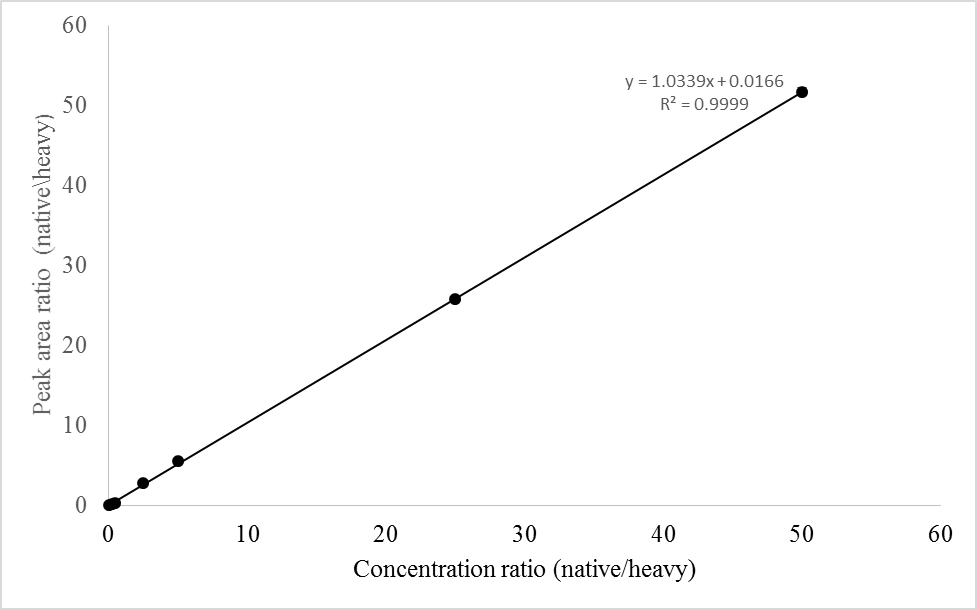


Fig 8. Calibration curve for DLTDAFLAEVEK (2D26)


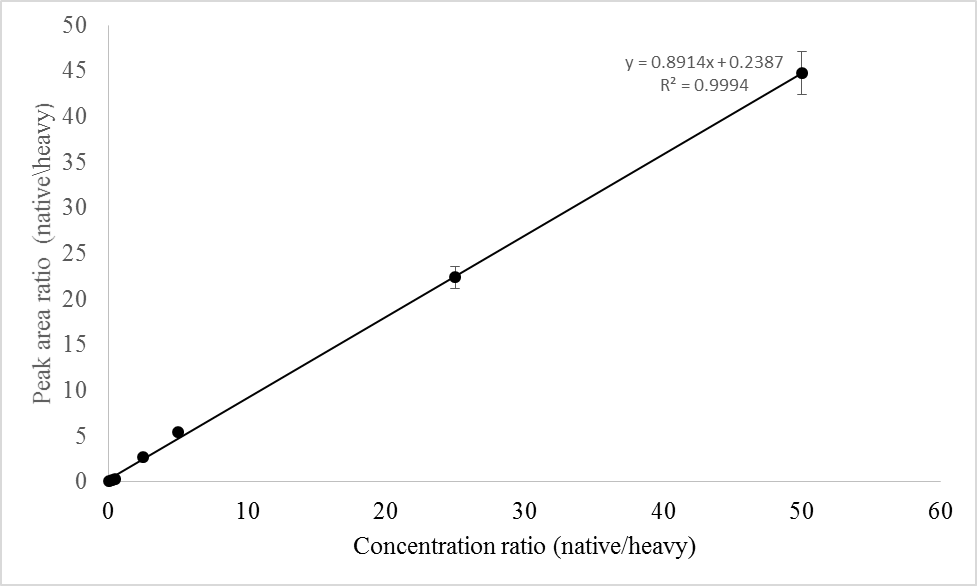


Fig 9. Calibration curve for DIDLSPVTIGFGSIPR (2E1)


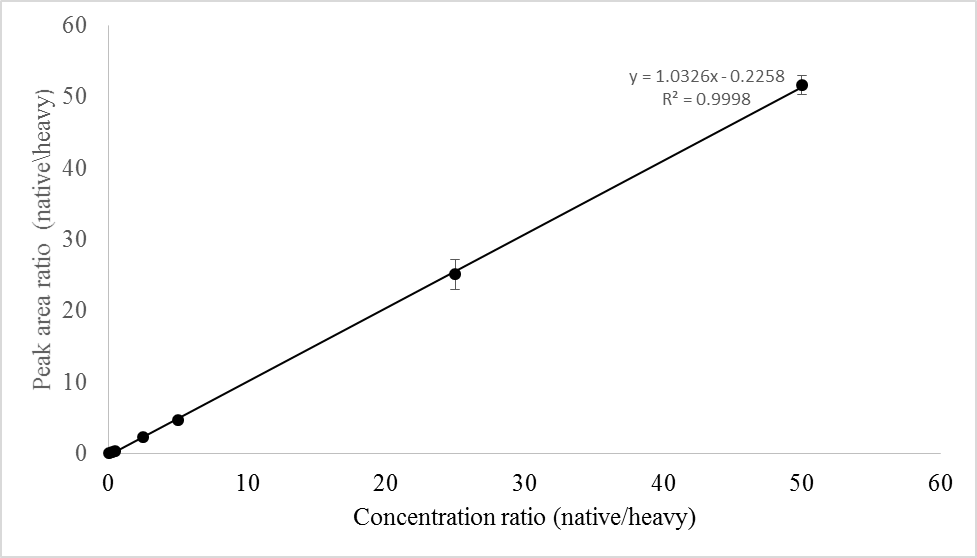


Fig 10. Calibration curve for ALLSPTFTSGK (3A11,41)


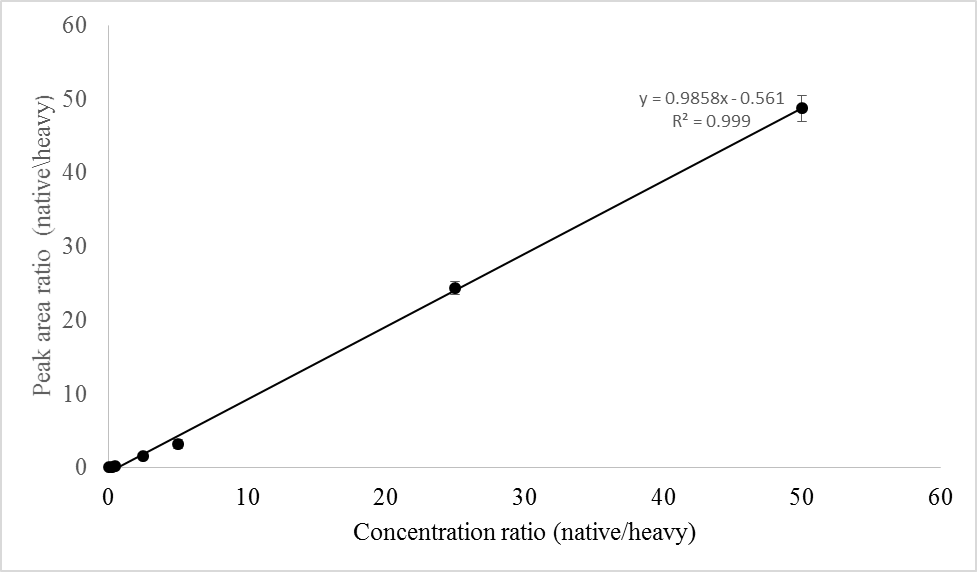


Fig 11. Calibration curve for GSIDPYVYLPFGNGPR (3A11,16)


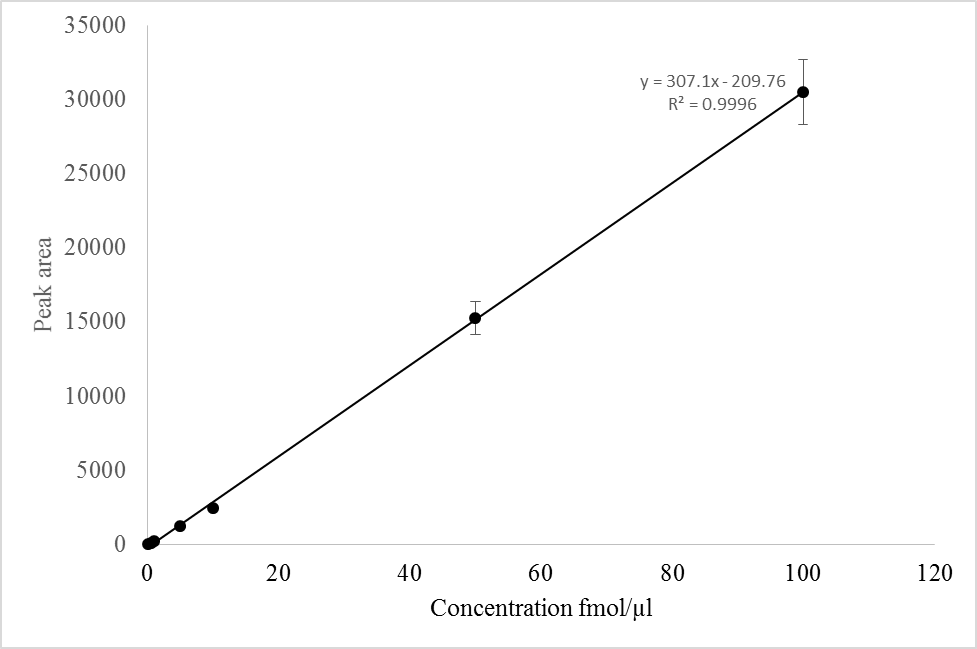


Fig 12. Calibration curve for NISQSFTNFSK (2C29)


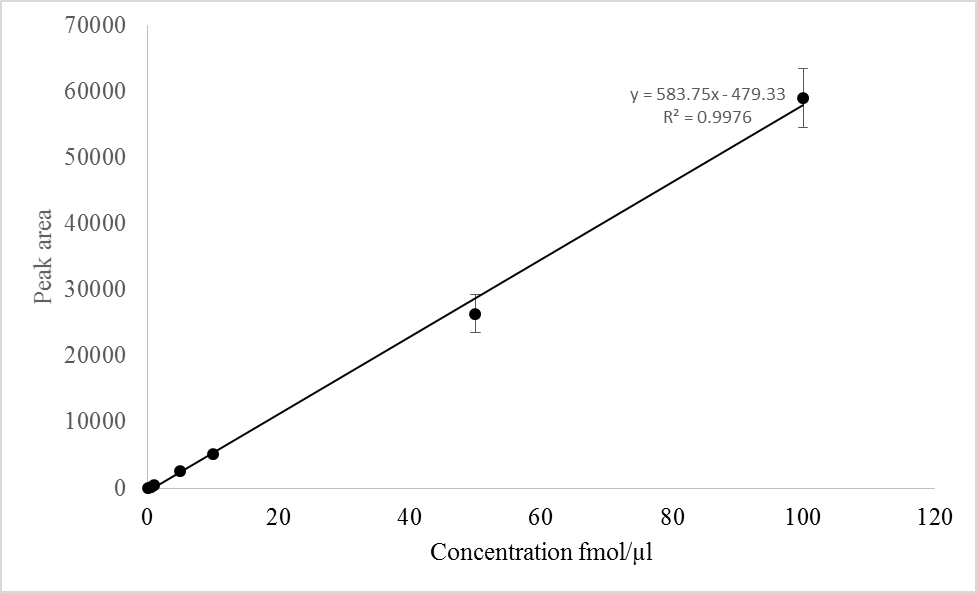


Fig 13. Calibration curve for MNMPYMDAVVHEIQR (2E1)


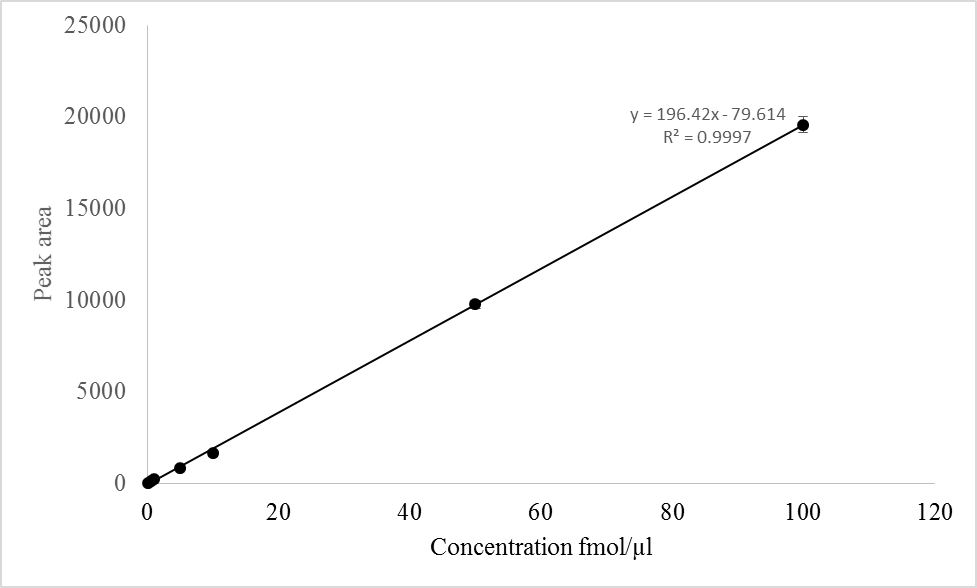


Fig 14. Calibration curve for FALMNMK (3A11,16)

**Тable A. Precision and accuracy of the CYP determinations.**

|  |  | 0.5 fmol/ µl | | 5 fmol/ µl | | 50 fmol/ µl | |
| --- | --- | --- | --- | --- | --- | --- | --- |
| CYP | Peptide | Accuracy % | CV % | Accuracy % | CV % | Accuracy % | CV % |
| 1А1 | VDMTPTYGLTLK | 88 | 14.5 | 101 | 2.9 | 105 | 3.9 |
| 1А2 | NSIQDITSALFK | 92 | 4.7 | 91 | 11.6 | 93 | 5.7 |
| 2С29 | NISQSFTNFSK | 111 | 8.4 | 95.1 | 5.0 | 98.8 | 7.2 |
| 2С29 | GTTVITSLSSVLHDSK | 104 | 7.4 | 91 | 14.5 | 94 | 2.1 |
| 2C39 | FINLVPNNIPR | 86.0 | 6.0 | 93.6 | 8.4 | 94.5 | 8.8 |
| 2C50,54 | VQEEIEHVIGK | 93.5 | 7.4 | 97.1 | 9.7 | 107.8 | 4.3 |
| 2D9 | FGDIVPVNLPR | 110 | 14.4 | 89.2 | 12.5 | 103.3 | 5.4 |
| 2D10 | FGDIAPLNLPR | 94.8 | 7.0 | 96.5 | 12.4 | 98.5 | 1.9 |
| 2D26 | DLTDAFLAEVEK | 92.3 | 4.1 | 110.5 | 4.5 | 103.3 | 1.0 |
| 2E1 | DIDLSPVTIGFGSIPR | 98.4 | 9.8 | 110 | 1.6 | 89.5 | 5.3 |
| 2E1 | MNMPYMDAVVHEIQR | 106 | 0.8 | 97 | 4.9 | 102 | 7.5 |
| 3A11,41 | ALLSPTFTSGK | 93.4 | 10.9 | 94 | 3.5 | 103 | 2.6 |
| 3A11,16 | GSIDPYVYLPFGNGPR | 89.1 | 8.4 | 103.8 | 9.1 | 97.4 | 3.6 |
| 3A11,16 | FALMNMK | 103.8 | 12.6 | 87 | 8.9 | 94.4 | 2.2 |
